# Dentoskeletal changes of long‐term oral appliance treatment in patients with obstructive sleep apnea: A systematic review and meta‐analysis

**DOI:** 10.1111/jopr.13946

**Published:** 2024-09-26

**Authors:** Yanlong Chen, Abdalgader I. Alhozgi, Fernanda R. Almeida

**Affiliations:** ^1^ Division of Orthodontics Department of Oral Health Sciences Faculty of Dentistry University of British Columbia Vancouver British Columbia Canada

**Keywords:** dentoskeletal changes, obstructive sleep apnea, oral appliance, side effects

## Abstract

**Purpose:**

This systematic review and meta‐analysis aimed to evaluate the dental and skeletal effects of the long‐term oral appliance (OA) treatment in patients with obstructive sleep apnea (OSA) and provide insights for clinicians in treatment planning and decision‐making for OSA patients undergoing OA treatment.

**Materials and Methods:**

A comprehensive literature search was conducted in major databases up to April 2024. Studies were included if they assessed long‐term OA treatment (≥6 months) in adults with OSA using any type of mandibular advancement device (MADs) or tongue retaining device (TRD). Dental and skeletal changes, measured by dental cast and cephalometric analysis, were the primary outcomes.

**Results:**

A total of 42 studies were included in the systematic review, with 23 included in the meta‐analysis. Long‐term OA treatment was associated with a significant decrease in overbite (0.87 mm, 95% CI: 0.69–1.05) and overjet (0.86 mm, 95% CI: 0.69–1.03). Subgroup analyses showed the decrease of overbite and overjet progressively changed over the years intervals. There was a significant retroclination of the upper incisors (U1‐SN, 2.58°, 95% CI: 1.07–4.08) and proclination of the lower incisors (L1‐MP, ‐2.67° (95% CI: ‐3.78–1.56). Skeletal changes were not significant.

**Conclusion:**

Overbite and overjet gradually decreased in the long‐term OA treatment, which might predominantly result from the retroclination of the upper incisors and the proclination of the lower incisors. The skeletal patterns in the anteroposterior and vertical direction might remain relatively stable over time. There was a tendency for the clockwise rotation of the mandible.

Obstructive sleep apnea (OSA) is characterized by periodic and repetitive, partial or complete collapse of the upper airway during sleep. Patients with OSA undergo reduced (hypopnea) or absent (apnea) airflow, associated with cortical arousal and blood desaturation.^1^ In the United States, OSA is present in approximately 25% of adults and is a major cause of excessive sleepiness.^2^, ^3^ The long‐term outcomes of OSA include cognitive dysfunction, hypertension, stroke, metabolic disease, and increased cardiovascular morbidity and mortality.^4^, ^5^, ^6^

Effective treatments for OSA include medical devices, behavioral measures, and surgery.^1^ Among all, positive airway pressure (PAP) is the primary therapy for individuals with symptomatic OSA of any severity,^7^ which normalizes AHI in more than 90% of patients while wearing the device.^8^, ^9^ However, PAP is associated with a low level of adherence.^10^, ^11^ According to the American Academy of Sleep Medicine (AASM) guidelines, the management of OSA patients by oral appliance (OA) is recommended for OSA patients who are intolerant to PAP or prefer an alternate therapy.^12^ OA treatment has also been effective for 50% to 70% of OSA patients with greater adherence, improving not only OSA symptoms but also a variety of physiologic and behavioral outcomes.^13^

Oral appliances can be divided into two main categories based on their mode of action.^14^ First and most common, mandibular advancement devices (MADs), consist of plates made to fit the upper and lower teeth.^13^, ^15^ Positions of these plates can be adjusted, allowing advancement of the mandible relative to the maxilla which could increase upper airway volume and reduce airway collapsibility.^16^, ^17^ Second, tongue retaining devices (TRDs), utilize suction pressure from the oral cavity to hold the tongue in an advanced position and prevent the tongue from falling back into the upper airway.^15^, ^17^

OA treatment has demonstrated both short‐term and long‐term side effects. Previously reported short‐term side effects include excessive salivation, mouth dryness, temporary occlusal changes, difficulty chewing, and discomfort in the gums, teeth, or jaws.^15^, ^18^, ^19^ Compared with the short‐term ones, the long‐term side effects of OA treatment are more progressive and irreversible.^17^ Pliska et al. observed a 1.9 mm reduction in overjet and a 2.3 mm reduction with OA treatment in a mean duration of 11 years.^20^ Despite these dental movements being mostly silent and unnoticed, they could lead to intolerance and discontinuation of treatment in some other patients. In clinical practice, it is crucial to inform patients about the long‐term side effects of OA treatment and monitor such changes regularly.^17^

Recent systematic reviews and meta‐analyses have extensively documented the dental and skeletal side effects of OA treatment.^21^, ^22^, ^23^, ^24^ Most studies indicated that the nature of these side effects is mainly dental instead of skeletal, and such reductions in overjet and overbite mainly resulted from the retroclination of the upper incisors and proclination of the lower incisors. However, these reviews have primarily focused on MADs, leaving a knowledge gap regarding the dentoskeletal changes associated with TRDs, which have been less explored.

Therefore, this systematic review aimed to provide updated evidence on the long‐term side effects of OA treatment, encompassing both MADs and TRDs, to facilitate treatment planning and decision‐making.

## METHODS

**FIGURE 1:**
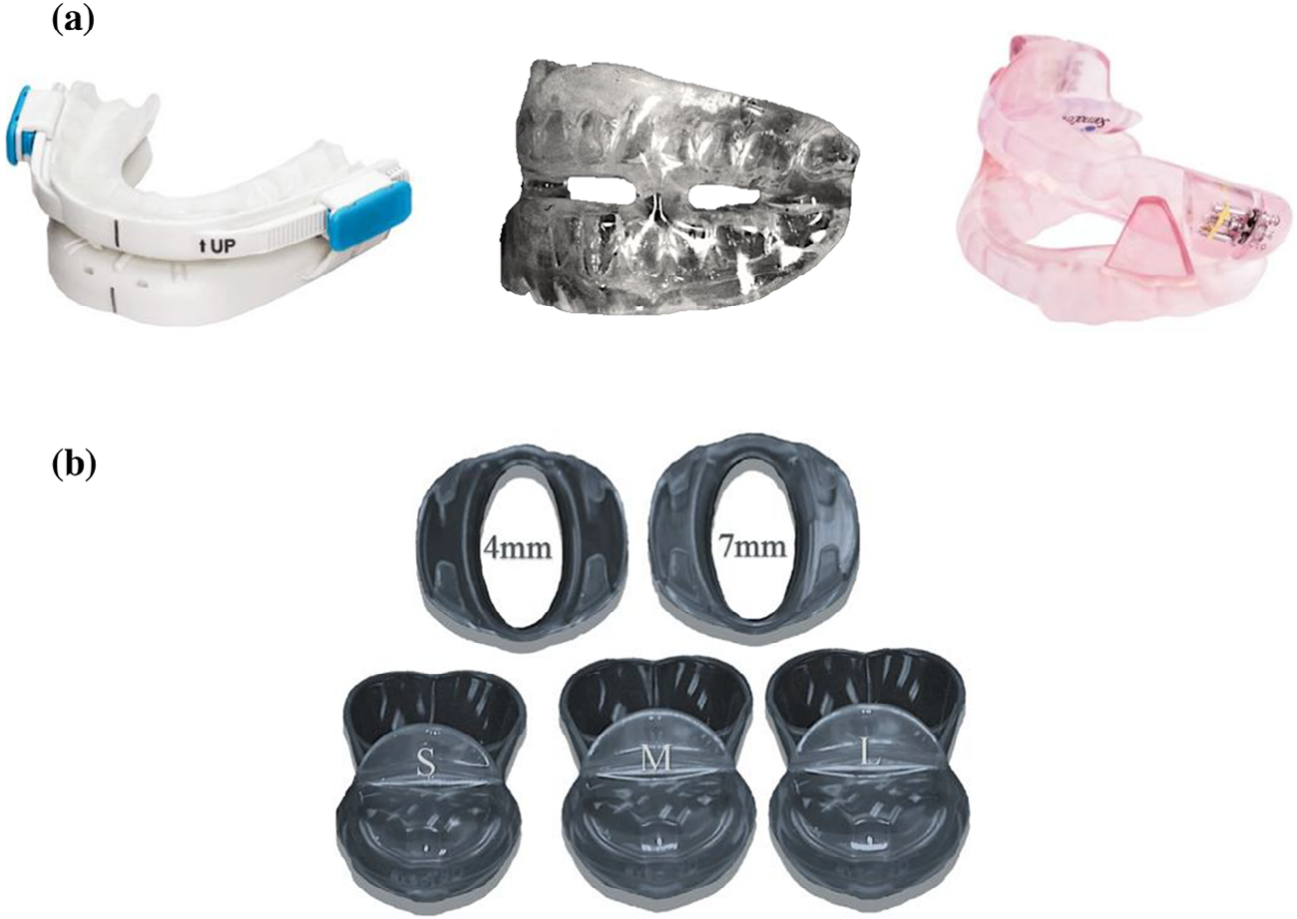
Different types of OAs. (a) MADs. (From left to right) Ready‐made, “boil and bite” device (BluePro); Custom‐made, monobloc device fabricated in dental labs; Custom‐made, bibloc device (SomnoDent Flex). (b) TSD.^29^ Three different sizes (small, medium, and large) and two titration accessories (4 and 7 mm). MAD, mandibular advancement devices; OA, oral appliance; TSD, tongue stabilizing device.

This systematic review and meta‐analysis was conducted according to the Preferred Reporting Items for Systematic Reviews and Meta‐Analyses (PRISMA) statement (2020 version).^25^ The following PICOS (Population/Intervention/Comparison/Outcomes/Study characteristic) elements were applied to determine study eligibility:

1. Population: Adults (age ≥ 18 years) diagnosed with OSA.
2. Intervention: Long‐term OA treatment (≥ 6 months). OAs include any specific type or design of MADs and TRDs (Figure 1). There were no restrictions on the materials used to make the OA, the titration process, or the method of fixation, allowing for a comprehensive assessment of the side effects of OAs.
3. Comparison: Before and after treatment self‐controlled comparison.
4. Outcome: Dental and skeletal changes, including dental cast analysis and cephalometric analysis.
5. Study design: Randomized and non‐RCTs, observational studies. All case reports and review articles were excluded.

### Literature search and study selection

The literature search was initially performed on March 6, 2023, and then updated on April 1, 2024. An extensive search of Ovid MEDLINE, Ovid Embase, and Web of Science was conducted using search terms in three domains: (“obstructive sleep apnea”, “oral appliances”, and “side effects” OR “dental and skeletal changes”). The detailed search strategies for each database are in the Supplementary Material (Table S1). Articles indexed in the databases with no restrictions on the date of publication and language of the article were searched. An additional grey literature search was conducted using Google Scholar in case of any other relevant published work (including conference abstracts, dissertations, clinical trial registries, etc.). Manual searching of the reference lists of previous systematic reviews was also conducted to increase the comprehensiveness of the search process.^21^, ^22^, ^23^, ^24^ The primary authors or the corresponding authors were contacted if needed. Results from the grey literature search will be carefully evaluated and compared with findings from published studies before study selection.

All the studies from the literature search were imported to the Covidence platform (Veritas Health Innovation, Melbourne, Australia. Available at www.covidence.org) for teamwork. Two investigators (Y.C. and A.A.) independently assessed studies based on their titles and abstracts. Studies that did not meet the inclusion were excluded. If a study could not be determined on the basis of title and abstract, the study was considered potentially relevant and the full text was obtained and reviewed. Any disagreement was resolved by discussing to a consensus or by reaching out to a third reviewer (F.A.).

### Critical appraisal

Two investigators (Y.C. and A.A.) independently conducted the critical appraisal of all the included studies, and any disagreements were resolved by discussion and consensus or by consulting a third reviewer (F.A.). Version 2 of the Cochrane risk‐of‐bias tool for randomized trials (RoB 2) was applied to assess the bias in the randomized controlled trials (RCTs),^26^ which includes five domains: bias arising from the randomization process, bias due to deviations from the intended interventions, bias due to missing outcome data, bias in the measurement of the outcome, and bias in the selection of the reported result. For each result, the risk of bias in each of the five domains was assessed by a series of signaling questions. The overall risk of bias was based on each domain and graded low, some concerns, or high.

Non‐randomized studies were assessed by the Risk of Bias in Non‐randomized Studies‐of Interventions (ROBINS‐I) tool.^27^ This tool comprises seven domains: bias due to confounding, bias in the selection of participants in the study, bias in the classification of interventions, bias due to deviation from intended interventions, bias due to missing data, bias in the measurement of outcomes, bias in the selection of the reported results. Similar to RoB 2, each of the seven domains was assessed by signaling questions, and the overall risk of bias was graded low, moderate, serious, or critical based on each domain.

### Data extraction

Data extraction was performed independently by two investigators (Y.C. and A.A.) using a standardized form developed for this systematic review and meta‐analysis. Discrepancies between reviewers were resolved through discussion or consultation with a third reviewer (F.A.) to achieve consensus. Study characteristics, including the author, year, study design, participants, baseline body mass index (BMI), baseline apnea–hypopnea index (AHI), baseline OSA severity, OA type, treatment duration, and measurements were extracted. Variables with the highest consistency between studies were chosen for the meta‐analysis. Other variables were then described as mean and standard deviation or median and interquartile range and were presented as descriptive only. Specifically, for studies that the standard deviations (SDs) were not provided, the SDs will be calculated by sample size and confidence intervals (CIs) using the methods in Cochrane Handbook.^28^

**FIGURE 2:**
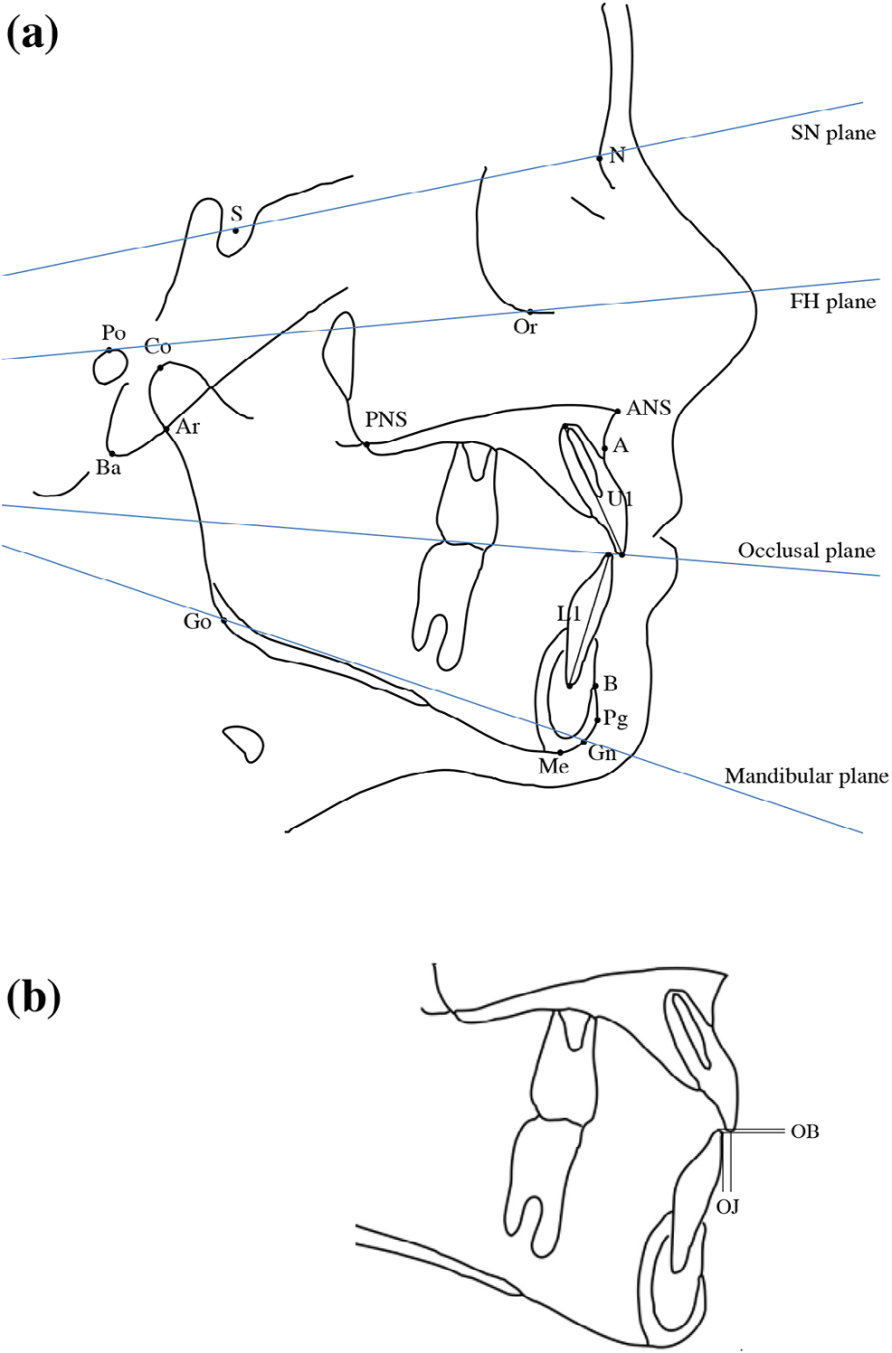
Sketch of variables in the cephalometric and dental cast analysis. (a) Cephalometric landmarks and reference planes. (1) Landmarks. (2) Dental measurements. OB (mm), the vertical overlap of U1 and L1; OJ (mm), the horizontal overlap of U1 and L1; U1‐SN (°), the angle between U1 and S‐N line; L1‐MP) (°), the angle between L1 and MP; U1‐L1 (°), the angle between U1 long axis and L1 long axis. (3) Skeletal measurements. SNA (°), the angle between S‐N‐A, SNB (°), the angle between S‐N‐B; ANB (°), the angle between A‐N‐B; MP‐SN (°), the angle between the mandibular plane and SN plane; MP‐FH (°), the angle between the mandibular plane and Frankfort (FH) plane. (b) Overjet and overbite. A, point‐A, ANS, anterior nasal spine; Ar, articulare; B, point‐B; Co, condylion; Gn, gnathion; Go, gonion; L1, lower incisor; Me, Menton; N, nasion; OB, overbite; OJ, overjet; PNS, posterior nasal spine; S, Sella; U1, upper incisor.

For meta‐analysis, the following variables were used as a measure of dental changes: overbite, overjet, U1‐SN (the angle between the long axis of the upper central incisor and the cranial base), L1‐MP (the angle between the long axis of the lower central incisor and the mandibular plane), U1‐L1 (the angle between the long axis of the upper central incisor and the long axis of the lower central incisor). The skeletal changes were measured by the following variables: SNA (the sagittal relationship of the maxilla to the cranial base), SNB (the sagittal relationship of the mandible to the cranial base), ANB (the sagittal relationship between the maxilla and mandible relative to the cranial base), MP‐SN (the angle between the mandibular plane and the cranial base), MP‐FH (the angle between the mandibular plane and the Frankfort plane).^29^ The definition of each variable is shown in Figure 2.

### Data synthesis

All eligible studies for meta‐analysis were imported into Review Manager (RevMan) Version 5.4 (The Cochrane Collaboration, 2020) to calculate the differences after treatment. The results of each study were weighed using the inverse variance method and tested for heterogeneity using the Chi‐square test and I^2^ statistics. If the I^2^ < 25%, the studies were considered homogenous, and a fixed‐effects model was used. If the I^2^ > 75%, the studies were regarded as highly heterogeneous, and a random‐effects model was applied. All results were visualized by forest plots with a 95% CI. To assess the risk of publication bias, funnel plots were generated for outcomes that had more than 10 eligible studies using the standard error and the difference of mean values.

Subgroup analyses were conducted to explore the sources of heterogeneity, including (1) study design: retrospective studies versus prospective studies; (2) risk of bias: low or moderate risk of bias versus high risk of bias; and (3) outcome measurements: dental cast analysis versus cephalometric analysis versus intraoral exams.

## RESULTS

**FIGURE 3:**
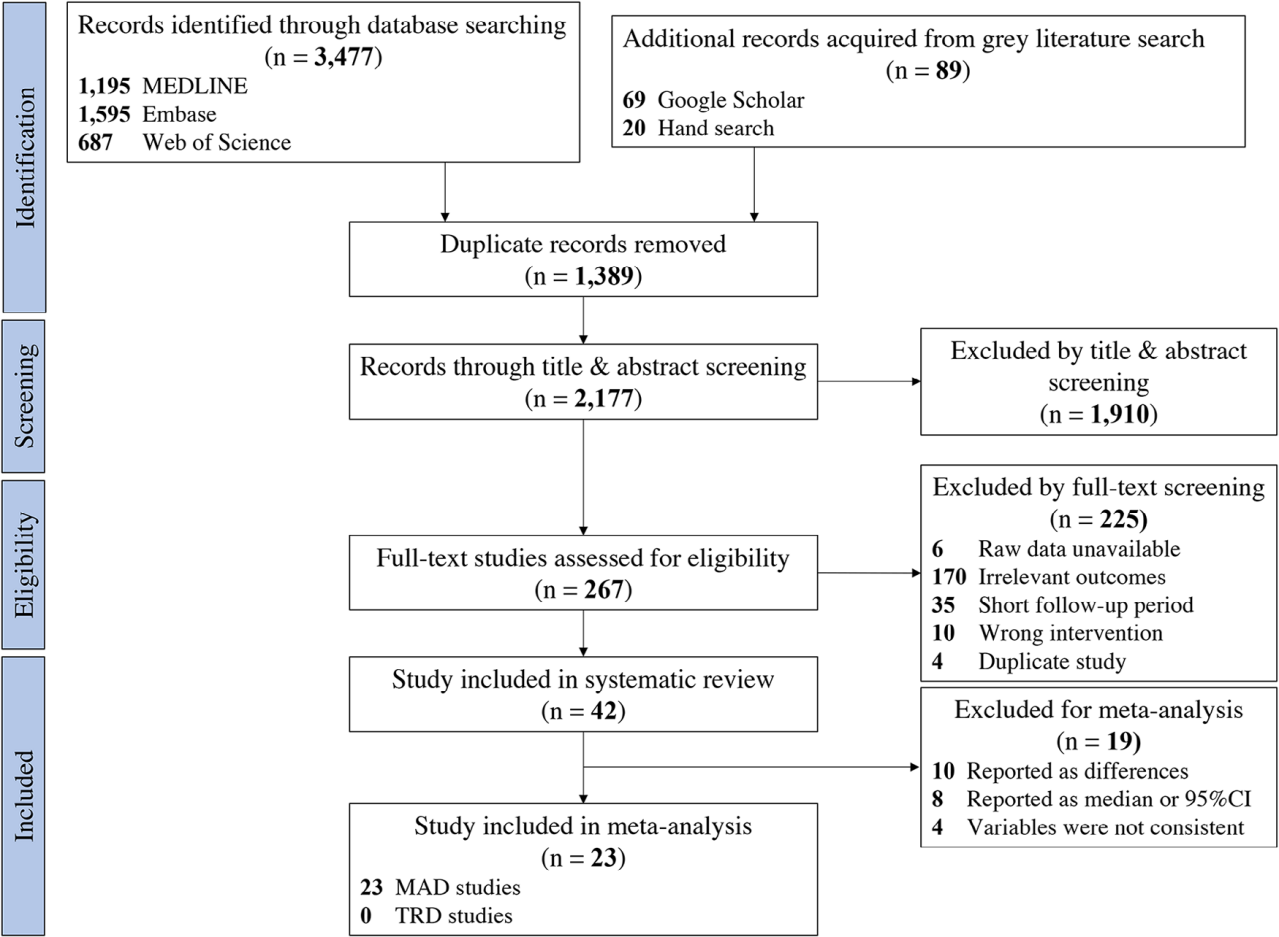
The flowchart of literature search and study selection. MAD, mandibular advancement devices; TRD, tongue retaining devices.

The study selection process is shown in Figure 3. The literature search generated 3566 records, including 1195 from Ovid MEDLINE, 1595 from Ovid Embase, 687 from Web of Science, and 89 from grey literature search (69 from Google Scholar and 20 from hand search). Following the removal of duplicates, 2177 records were screened by titles and abstracts. Among them, 267 records remained and were eligible for full‐text screening. Then, 225 records were excluded, leaving 42 studies in qualitative synthesis, which consisted of 4 RCTs and 38 non‐RCTs. The detailed characteristics of the included 42 studies are shown in Table 1.

**TABLE 1:** Characteristics of eligible studies.

Author	Year	Study design	Participants (male / female)	Baseline age (y, mean ± SD or specified)	Baseline BMI (kg/m^2^, mean ± SD or specified)	Baseline OSA severity	OA type	Treatment duration (year or month, mean ± SD or specified)	Measurement method	Outcomes
**MADs**										
Bondemark^59^	2000	Prospective	32 (23/9)	54.4 ± 8.78	N/A	AHI: 27 ± 19, range [5–74]	Monobloc	2 y	Dental casts analysis	OB, OJ
Fritsch, et al^48^	2001	Prospective	22	Median 49.5, IQR [44.0–58.0]	26.3, range [25.4–28.4]	AHI: 27.6 ± 3.5	Monobloc, Herbst	Median 14 m, IQR [12–30] m	Cephalometric analysis, Dental casts analysis	OB, OJ, SNA, SNB, ANB, L1‐MP
Marklund, et al^38^	2001	Retrospective	92	53.5 ± 8.5	N/A	5 ≤ AHI ≤ 20: *n* = 46, AHI > 20: *n* = 28	MAD made of soft elastomer	2.5 ± 0.5 y	Dental casts analysis	OB, OJ
Robertson^39^, ^40^	2001,2003	Retrospective	100 (87/13)	49.3 ± 8.5, range [33–74]	N/A	N/A	Non‐adjustable rigid splint	6, 12, 18, 24 or 30 m	Cephalometric analysis	OB, OJ, SNA, SNB, ANB, L1‐MP
Fransson^51^, ^52^	2002,2004	Prospective	65	55, range [31–73]	29.2 ± 3.6, range [21–38]	N/A	Mandibular protruding device	2 y	Cephalometric analysis, intraoral exams	OB, OJ, SNA, SNB, U1‐L1
Rose^64^	2002	Retrospective	34	52.9 ± 9.6, range [27.1‐64.6]	28.6 ± 4.2	AHI: Median 21.7, IQR [14.9‐28.4]	Hard acrylic and joined by U‐shaped clasps	29.6 ± 5.1 m, range [24.1‐43.5] m	Cephalometric analysis, Dental casts analysis	OB, OJ, SNA, SNB, ANB, U1‐SN, L1‐MP, U1‐L1, MP‐SN
Ringqvist^33^	2003	Prospective	45	48.9, 95% CI: (46.3–51.3)	27.0, 95% CI: (25.7–28.2)	5 < AI < 25	MAD	4.1y, 95% CI; (4.0‐4.2) y	Cephalometric analysis	OB, OJ, MP‐SN
Tegelberg^34^	2003	Prospective	74	50% group: 51.8, 95% CI: (49.0‐54.6); 75% group: 54.4, 95% CI: (52.4‐56.4)	50% group: 27.4, 95% CI: (26.4‐28.4); 75% group: 27.9, 95% CI: (26.6‐29.3)	AHI: 50% group: 16.2 ± 2.9; 75% group: 18.9 ± 4.7	One‐piece heat‐cured acrylic polymer	1 y	Intraoral exams	OB
Battagel^82^	2005	Retrospective	30 (26/4)	52.2, range [34.0–73.4]	25.7, range [20.9–36.9]	N/A	Herbst MAS	3.6 y, range [2.2–6.1] y	Cephalometric analysis	OB, OJ
Almeida^67^, ^68^	2006	Retrospective	71 (63/8)	49.7 ± 9.7	29.3 ± 5.9	RDI: 28.9 ± 17.0	Klearway	7.3 ± 2.1 y	Cephalometric analysis	OB, OJ, U1‐SN, L1‐MP, MP‐SN
Hou^73^	2006	Prospective	67 (50/17)	46.9 ± 8.88, range [20.5–71.4]	N/A	N/A	Modified Harvold monobloc type of functional appliance	T0–T1: 1.0 ± 0.16 y, range [0.7–1.5] y; T0–T2: 2.0 ± 0.22 y, range [1.6–2.6] y; T0–T3: 3.0 ± 0.2 y, range [2.5–3.6] y	Cephalometric analysis	OB, OJ, SNA, SNB, ANB, MP‐SN
Marklund^41^	2006	Prospective	155(127/28)	Median 51, IQR [22–74]	Median 27, IQR [19–42]	AHI: Median 13, IQR [0–76]	Soft elastomeric device and Hard acrylic device	5.4 ± 0.8 y	Dental casts analysis	OB, OJ
Hammond^42^	2007	Retrospective	46 (34/12)	49.4 ± 9.82, range [29.4–72.5]	29.1 ± 4.99, range [19.9–42.4]	AHI: 25.3 ± 17.74, range [3.0–81.0]	2‐piece acrylic design with full occlusal coverage and a screw	25.1 ± 11.8 m	Cephalometric analysis, Dental casts analysis	OB, OJ, SNA, SNB, ANB, U1‐SN, L1‐MP, U1‐L1, MP‐SN
Chen^43^, ^44^	2008	Retrospective	70 (62/8)	50.0 ± 9.6, range [31–81]	29.3	RDI: 28.0, range [0.0–68.0]	Klearway	88.4 ± 26.7 m	Dental casts analysis	OB, OJ, Arch length, AW, CS, Anteroposterior relationship
Ghazal^53^	2008	Retrospective	21 (17/4)	50.3 ± 11.6, range [32.6–67.2]	26.4 ± 2.7	N/A	Thornton adjustable positioner	33 ± 9 m	Dental casts analysis	OB, OJ
Doff^35^, ^36^	2010,2013	Retrospective	51 (43/8)	49 ± 10	32 ± 6	AHI: 39 ± 31	Thornton Adjustable Positioner	2.3 ± 0.2 y range [2.1–3.1] y	Cephalometric analysis	OB, OJ, SNA, SNB, ANB, L1‐MP, U1‐L1, MP‐SN
Marklund^60^	2010	Prospective	10	Mean 51, IQR [32–64]	Mean 28, IQR [23–37]	AHI: 10, range [1.6–19]	A monobloc elastomeric appliance	2.3 years, range [2.2–2.4] years	Dental casts analysis	OB, OJ, SNA, SNB
Martínez‐Gomis^54^	2010	Prospective	40	54.1 ± 8.7, range [35–70]	N/A	AHI > 10	MAD consisted of two full‐coverage	4.8 y, range [3.6–5.8] y	Intraoral exams	OB, OJ
Gong^56^, ^57^	2011,2013	Retrospective	25	N/A	N/A	Short‐term: 24.50, range [14.65–54.05], Long‐term: 25.55, range [11.71–43.65]	Mandibular repositoner	Median: 33 m, IQR [24–103] m	Cephalometric analysis	OB, OJ, SNA, SNB, ANB, U1‐SN, L1‐MP, U1‐L1, MP‐SN
Vezina^45^	2011	Prospective	57 (48/9)	Traction group: 56.1 ± 8.9; Compression group: 54.1 ± 10.0; Control group: 57.1 ± 8.7	Traction group: 24.4 ± 2.8; Compression group: 26.7 ± 2.9; Control group: 28.5 ± 2.7	AHI: Traction group: 31.2 ± 16.8; Compression group: 30.8 ± 21.4; Control group: 40.8 ± 42.7	Traction group: traction‐based device; Compression group: compression‐based device	Traction group: 3.7 ± 1.2 y; Compression group: 3.6 ± 1.2 y	Cephalometric analysis	U1‐SN, L1‐MP, MP‐SN
Pliska^20^	2014	Retrospective	77 (62/15)	47.5 ± 10.2, range [26–70]	29.4 ± 7.2, range [18.7–63.6]	AHI: 29.8 ± 16.9, range [2.4–77.4]	Klearway	11.1 ± 2.8 y	Dental casts analysis	OB, OJ, Crowding, Intercanine width, Intermolar width
Geoghegan^37^	2015	Prospective	45 (34/11)	52, range [27–79]	N/A	AHI: 21.1, range [14.2−50.1]	Twinblock, monobloc	12 w each with 2 w wash‐out	Cephalometric analysis	OB, OJ, SNA, SNB, ANB
Wang^58^	2015	Prospective	42 (31/11)	47 ± 10, range [26–70]	N/A	AHI:27 ± 19, range [5–74]	Silensor	4 ± 3 y, range [1–11] y	Cephalometric analysis	OB, OJ, SNA, SNB, ANB, U1‐SN, L1‐MP, U1‐L1, MP‐FH
Marklund^61^	2016	Prospective	9 (8/1)	Median 68.1, IQR [60.0–76.3]	Median 26.5, IQR [24.7–31.1]	AHI: Median 26.5, IQR [24.7–31.1]	Custom‐made device	Median 16.5 y, IQR [16.3–18.0] y	Intraoral exams	OB, OJ
Alessandri‐Bonetti^70^	2017	Prospective	20 (15/5)	57 ± 11.4	26.5 ± 3.6	AHI: 19.1 ± 14.5	Silensor	3.5 ± 1.1 y	Cephalometric analysis, Dental casts analysis	OB, OJ, SNA, L1‐MP, MP‐SN
Fransson^83^	2017	Prospective	41 (31/10)	N/A	N/A	N/A	Non‐adjustable monobloc	10 y	Dental casts analysis	OB, OJ, intercanine width, Intermolar width, Dental arch depth
Knappe^84^	2017	Prospective	43 (30/13)	Median 54, IQR [26–73]	N/A	AHI: Short‐term: 22.8 ± 10.8; Long‐term: 20.6 ± 8.9	Adjustable two‐piece, custom‐made, hard acrylic MAD with full occlusal coverage	T2: 1 y; T3: 3 y	CBCT, Intraoral exams	OB, OJ
Norrhem^46^	2017	Prospective	41 (29/12)	Flex group: Median 61.65, IQR [55.80–66.78] Rigid group: Median 60.80, IQR [56.00‐66.00]	N/A	AHI: Flex group: Median 15.00, IQR [10.25–21.00]; Rigid group: Median 12.00, IQR [6.00‐21.50]	Flex group: Narval; Rigid group: SomnoDent	Median 2.9 y, IQR [2.7–3.1] y	Dental casts analysis	OB, OJ, Little's Index upper/lower, Intercanine width
Araie^21^	2018	Retrospective	64 (44/20)	57.7 ± 14.2	23.9 ± 3.6	AHI: 24.9 ± 14.7	Acrylic monoblock	4.3 ± 2.1	Cephalometric analysis	OB, OJ, U1‐SN, L1‐MP, U1‐LI, SNA, SNB, ANB, MP‐SN
Teixeira^72^	2018	Prospective	15 (9/6)	47.23 ± 9.70	31.41 ± 4.49	AHI: 22.09 ± 5.99	Twinblock	6.47 ± 2.01 y	Cephalometric analysis, Dental casts analysis	OB, OJ, L1‐MP, Intermolar distance, Intercanine distance
Hamoda^65^	2019	Retrospective	62 (52/10)	49.0 ± .6, range [29–65]	29.1 ± 6.9, range [18.7–63.6]	AHI: 30.0 ± 14.6, range [7.4–66.2]	Klearway, SomnoDent	12.6 ± 3.9 y, range [8.0–21.4] y	Cephalometric analysis	SNA, SNB, U1‐SN, L1‐MP, MP‐SN, MP‐FH
Vigié Du Cayla^49^	2019	Prospective	24 (15/9)	54.3 ± 12.6	27.2 ± 5.7	AHI: 35.5 ± 18.2	SomnoDent, ORM	3.9 ± 2.4 y	Cephalometric analysis	Maxillary central incisor inclination, Mandibular central incisor inclination, Sagittal position of the maxilla and mandible
Fransson^71^	2020	Prospective	45 (35/10)	54 ± 8.0, range [37–73]	29 ± 3.8, range [21‐36]	For snoring, ODI < 5; For OSA, ODI ≥ 5	Non‐adjustable monobloc	10 y	Cephalometric analysis	SNA, SNB, U1‐SN, L1‐MP
Hu^55^	2020	Retrospective	18	39.94 ± 12.71, range [22–73]	24.85 ± 2.27	AHI: 26.93 ± 19.28	Adjusted activator appliances	6.57 ± 1.98 y	Dental casts analysis	OB, OJ
Marklund^62^	2020	Retrospective	38 (26/12)	Median 64.0, IQR [56.7–68.8]	N/A	AHI: Median 10.0, IQR [4.5–22.8]	Monobloc, SomnoDent, or Narval	Median 9.5 y, IQR [5.8‐14.3] y	Dental casts analysis	OB, OJ
Uniken Venema^63^	2020	Prospective	14 (12/2)	61 ± 8	32.4 ± 6.6	AHI: 31.7 ± 20.6	Thornton Adjustable Positioner	2 y, 10y	Dental casts analysis	OB, OJ
Heda^50^	2021	Retrospective	21 (15/6)	49.5 ± 11.8	26.7	AHI:16.6	Klearway, SomnoDent	7.9 ± 3.3 y	Cephalometric analysis, Dental casts analysis	OB, OJ, ANB, L1‐MP, MP‐SN, MP‐FH
Baldini^47^	2022	Prospective	117 (81/36)	62.0, range [54.0–69.0]	26.0, range [24.0–28.0]	AHI: 29.3 ± 13.8	AMO device, SomnoDent	56.0 m, range [32.0−80.0] m	Cephalometric analysis	OB, OJ, SNA, SNB, ANB, L1‐MP
Zheng^66^	2023	Prospective	23 (6/17)	50.5 ± 14.2 [26.4–73.9]	23.0 ± 3.8, range [18.0–33.1]	23.3 ± 13.2, range [6.4–54.6]	SomnoDent	3.1 ± 1.4 y, range [1.2−6.3] y	Cephalometric analysis	SNA, SNB, ANB, U1‐SN, L1‐MP, MP‐SN
**TRDs**										
Chen^30^	2008	Retrospective	5 (2/3)	46.2 ± 12.3	N/A	AHI (*n* = 2): 26.4–33.3	TRD	6.1 ± 3.5 y, range [2.7–10.1] y	Dental casts analysis	OB, OJ, Arch width, Spee's curve depth, Anteroposterior relationship
Eid^32^	2016	Prospective	10	37.6 ± 10.1	N/A	AHI > 5	TRD	6 m	Dental casts analysis	OB, OJ, Occlusal contact points, Anteroposterior movement
Alshhrani^31^	2024	Prospective	6 (5/1)	55.1 ± 10.3	26.3 ± 2.4	RDI: 14.0 ± 10.7	TRD	30 m	Dental casts analysis	Arch width, Arch length, Maxillary/mandibular overlap reference curve, Spee's curve depth, Occlusal plane changes

Abbreviations: AHI, apnea– hypopnea index; ANB, the sagittal relationship between the maxilla and mandible relative to the cranial base; AW, arch width; BMI, body mass index; CI, confidence interval; IQR, interquartile range; L1‐MP, the angle between the long axis of the lower central incisor and the mandibular plane; m, month; MAD, mandibular advancement device; MP‐SN, the angle between the mandibular plane and the cranial base; MP‐FH, the angle between the mandibular plane and the Frankfort plane; OA, oral appliance; OB, overbite; OJ, overjet; OSA, obstructive sleep apnea; RDI, respiratory disorder index; SNA, the sagittal relationship of the maxilla to the cranial base; SNB, the sagittal relationship of the mandible to the cranial base; SD, standard deviations; TRD, tongue retaining devices; U1‐L1, the angle between the long axis of the upper central incisor and the long axis of the lower central incisor; U1‐SN, the angle between the long axis of the upper central incisor and the cranial base; w, week; y, year.

Among the 42 studies, 23 were selected to perform meta‐analysis, while 19 were excluded from meta‐analysis and were presented in systematic review only. The variables of all three TRDs studies were not consistent and thus did not allow for systematic review.^30^, ^31^, ^32^ The results of each excluded study and the reason for exclusion are summarized in the appendix table (Table S2). Subgroup analyses were conducted for overbite and overjet based on treatment duration and were categorized into four subgroups: 6–12, 12–24 m, 24–36 m, and more than 36 m.

Funnel plots of the standard error for OB, OJ, L1‐MP, SNA, SNB, and ANB were attached in the appendix (Figure S1). All the six funnel plots were considered symmetrical, indicating a limited or low risk of publication bias.

**FIGURE 4:**
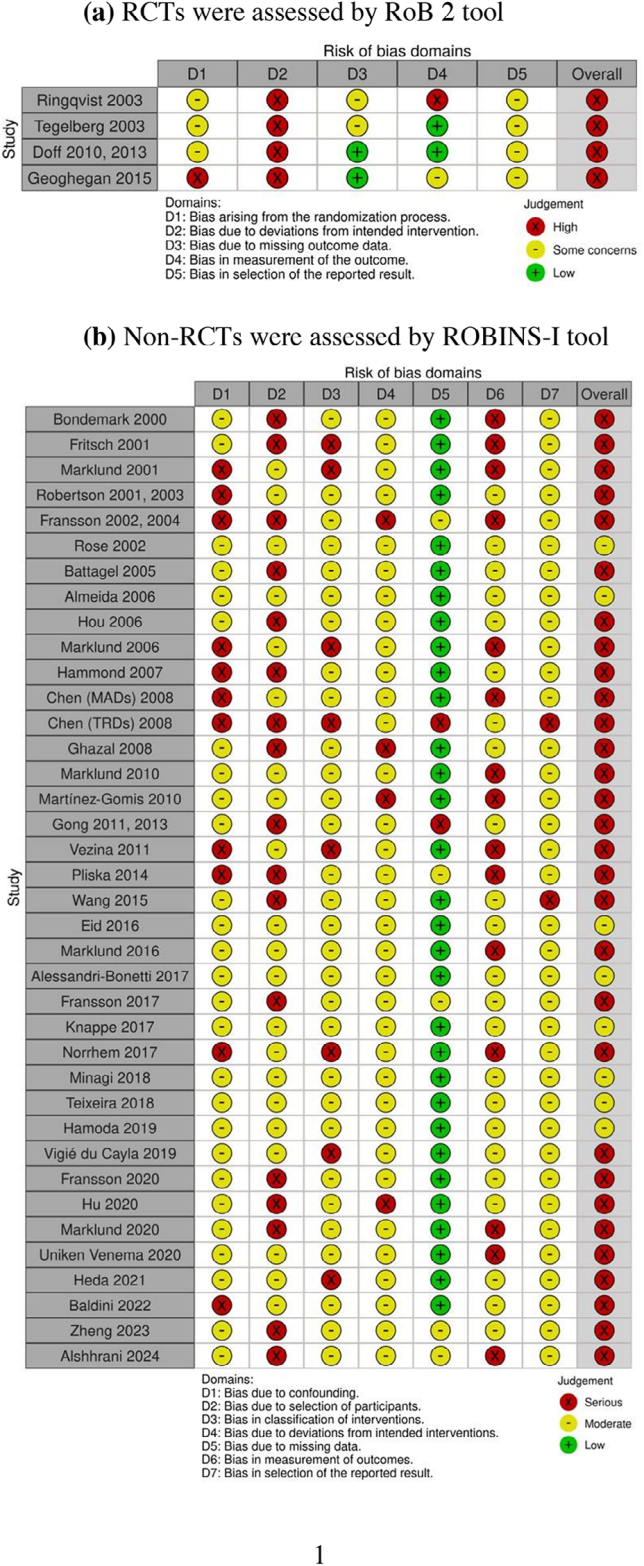
Results of the critical appraisals. (a) RCTs were assessed by the RoB 2 tool. (b) Non‐RCTs were assessed by ROBINS‐I tool. RCT, randomized controlled trials; RoB 2, risk‐of‐bias tool; ROBINS‐I, risk of bias in non‐randomized studies‐of interventions.

The results of the critical appraisal for individual studies are shown in Figure 4. For RCTs, all four studies were assessed to be high risk due to deviations from intended interventions, since it was hard to blind the participants of the groups (e.g., wear or not wear, OAs or CPAP).^33^, ^34^, ^35^, ^36^ One study has high risk in the randomization process,^37^ and one study has major limitations in the measurement of the outcomes.^33^

For non‐RCTs, 30 out of 38 studies were assessed to have a serious risk of bias. Among all the domains that have a serious risk of bias, 11 out of 38 studies were due to poor reliability or validity of the measurement methodology,^20^, ^30^, ^38^, ^39^, ^40^, ^41^, ^42^, ^43^, ^44^, ^45^, ^46^, ^47^ 17 out of 38 were due to selection of participants, and 12 out of 38 were due to classifications of intervention^30^, ^38^, ^41^, ^45^, ^46^, ^48^, ^49^, ^50^ or deviation from the intended intervention.^51^, ^52^, ^53^, ^54^, ^55^ Only two studies reported missing data,^30^, ^56^, ^57^ and two studies were considered to have a serious risk of bias due to the selective reporting from multiple analyses.^30^, ^58^ For the measurement of outcomes, 14 out of 38 showed serious risk of bias.^20^, ^31^, ^38^, ^41^, ^43^, ^44^, ^45^, ^46^, ^48^, ^51^, ^52^, ^54^, ^59^, ^60^, ^61^, ^62^, ^63^ The major concern was the subjective measurement (i.e., evaluators were not blinded to the groups, lack of inter‐evaluator verification, and evaluators were vulnerable to influence by knowledge of the intervention received by study participants).

### Dental changes of long‐term OA treatment

All included studies showed that both OB and OJ decreased after long‐term OA treatment, except for one study by Tegelberg et al.,^34^ which found a 0.1 mm increase in overbite after 1 year of MAD treatment. However, only 26 participants were included in that study group, and no statistical significance was found. As for the angle of upper incisors, six studies showed a significant decrease in the U1‐SN angle,^52^, ^56^, ^57^, ^58^, ^64^, ^65^, ^66^ while four studies had no statistical significance of the change.^42^, ^45^, ^67^, ^68^, ^69^ For the lower incisors, 13 studies demonstrated a significant increase of the L1‐MP angle,^35^, ^36^, ^39^, ^40^, ^42^, ^47^, ^50^, ^56^, ^57^, ^58^, ^64^, ^65^, ^66^, ^70^, ^71^ while only three studies showed no statistical significance.^45^, ^48^, ^72^ For the angle between upper and lower incisors, three studies showed a significant decrease of the U1‐L1 angle,^35^, ^36^, ^42^, ^51^, ^52^ while four studies had no statistical significance.^56^, ^57^, ^58^, ^64^

In meta‐analysis, the forest plots of OB, OJ, U1‐SN, L1‐MP, and U1‐L1 along with the mean difference and 95% CI are shown in Figure 5. The results of subgroup analyses were detailed in the Supplementary Material (Figure S2), which showed that prospective studies demonstrated more homogenous results compared to retrospective studies.

FIGURE 5The forest plots of dental changes in the long‐term OA treatment. (a) OB. (b) OJ. (c) The angle between the long axis of the upper central incisor and the cranial base (U1‐SN). (d) The angle between the long axis of the lower central incisor and the mandibular plane (L1‐MP). (e) The angle between the long axis of the upper central incisor and the long axis of the lower central incisor (U1‐L1). OA, oral appliance; OB, overbite; OJ, overjet.

**Figure:**
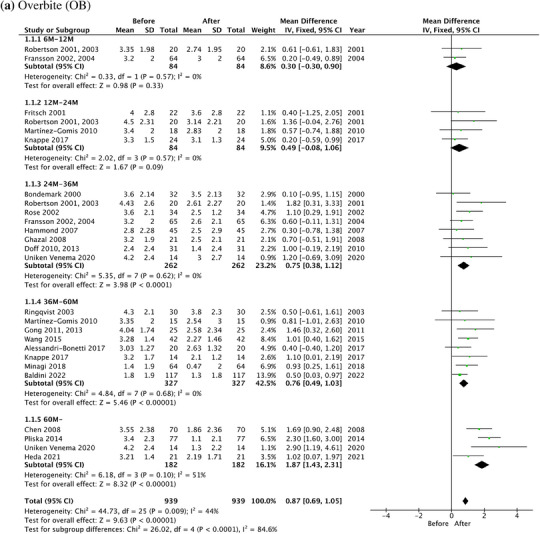

**Figure:**
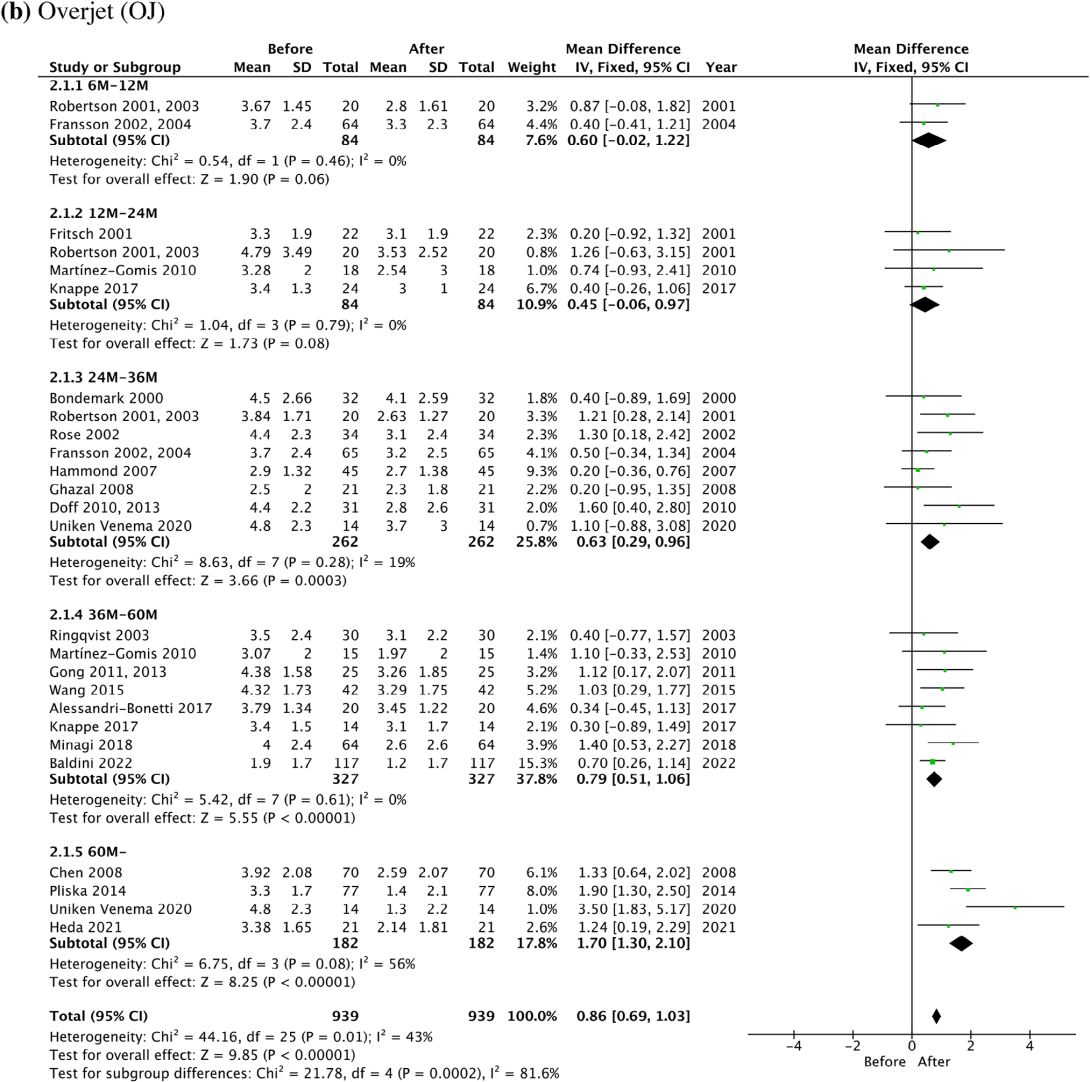

**Figure:**
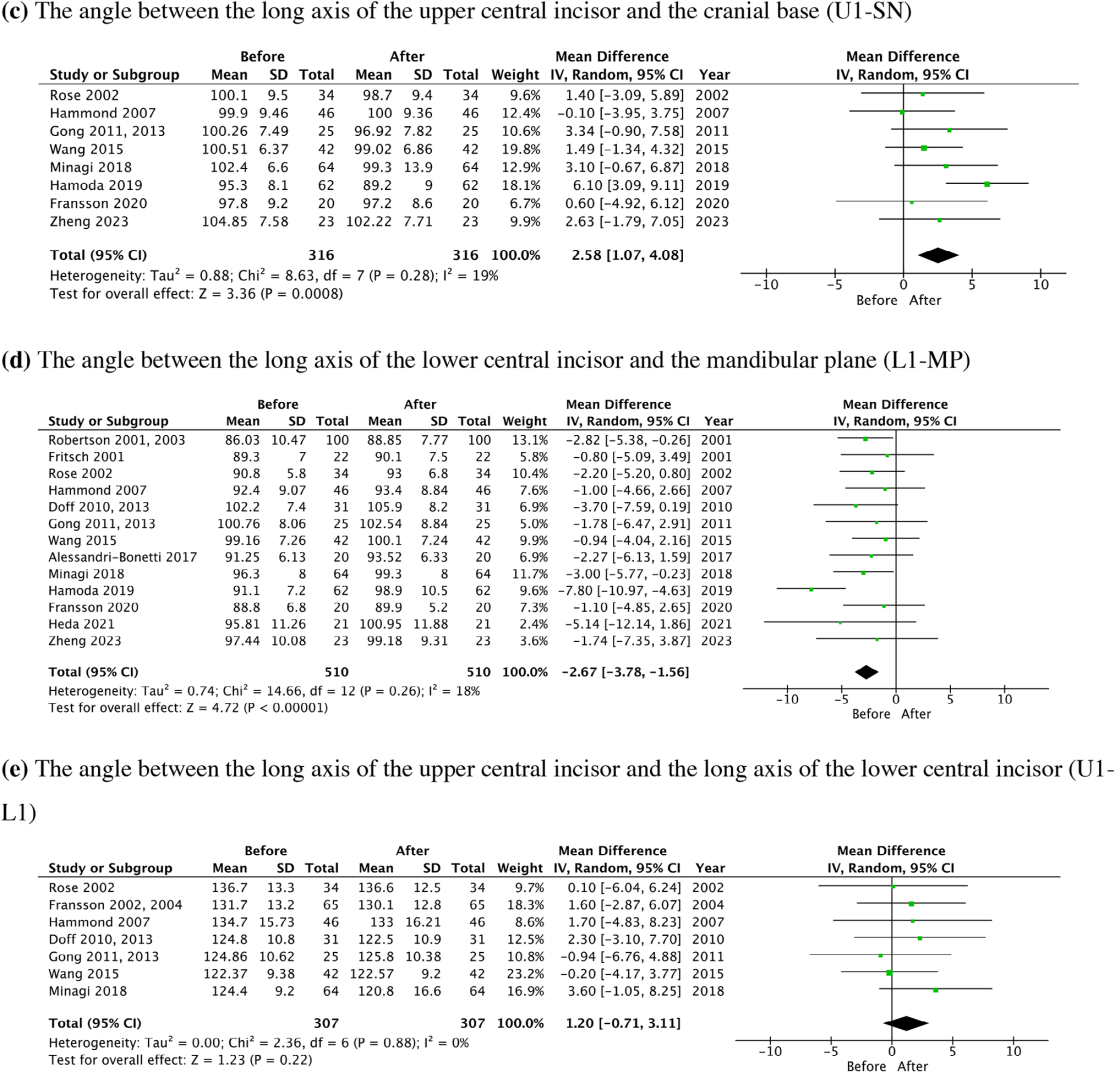

Overall, the total decrease in OB was 0.87 mm (95% CI: 0.69–1.05, *p* < .00001), and the total decrease in OJ was 0.86 mm (95% CI: 0.69–1.03, *p* < .00001) after the long‐term OA treatment. The overall I^2^ statistics for OB and OJ were 44% and 43 %, showing a moderate heterogeneity between different studies.

Subgroup analyses showed the decrease of OB over the years intervals was 0.30 mm (95% CI: ‐0.30–0.90, *p* = .33), 0.49 mm (95% CI: ‐0.08–1.06, *p* = .09), 0.75 mm (95% CI: 0.38–1.12, *p* < .0001), 0.76 mm (95% CI: 0.49–1.03, *p* < .00001), and 1.87 mm (95% CI: 1.43–2.31, *p* < .00001), at 6—12 m, 12—24 m, 24—36 m, 36—60 m and more than 60 m, respectively. The decrease of OJ was 0.60 mm (95% CI: ‐0.02–1.22, *p* = .06), 0.45 mm (95% CI: ‐0.06–0.97, *p* = .08), 0.63 mm (95% CI: 0.29–0.96, *p* = .0003), 0.79 mm (95% CI: 0.51–1.06, *p* < .00001), and 1.70 mm (95% CI: 1.30–2.10, *p* < .00001), at 6—12 m, 12—24 m, 24—36 m, 36—60 m and more than 60 m, respectively. The test for subgroup differences for OB and OJ was 84.6% (*p* < .00001) and 81.6% (*p* = .0002), respectively, indicating that the treatment effects significantly increased over time.

The total change of U1‐SN and L1‐MP was 2.58° (95% CI: 1.07–4.08, *p* = .0008) and ‐2.67° (95% CI: ‐3.78–1.56, *p* < .00001), respectively. There is no statistical significance of the total change of U1‐L1, which was 1.20° (95% CI: ‐0.71–3.11, *p* = .22).

For the TRDs, all three included studies were based on dental cast analyses. Eid et al. found that OB and OJ decreased by 0.2 ± 0.5 mm and 0.2 ± 0.6 mm after 6 months of treatment without statistical significance.^32^ There was also no significant change in the occlusal contact points in the cuspid‐incisor region or the premolar–molar region.^32^ Chen et al. observed that OB and OJ decreased in almost the entire arch after 76 months of TRD use,^30^ and both tended to decrease more on anterior teeth than posterior teeth.^30^ There was also a tendency of posterior open bite over time. The lower incisors tended to move forward, resulting in the relief of crowding in the lower anterior section. Other dental arch morphology changes include the depth of the CS on both sides becoming shallower, and the greatest arch width changes were noticed in the inter‐first molar for both the maxilla and mandible.^30^ Alshhrani et al. reported the range of teeth movements was between 0.1 and 1.1 mm after an average duration of 31.1 months.^31^ The observed changes were mainly in dental arch width and length variables, demonstrating an overall enlargement of upper and lower arches.^31^ The average cloud‐to‐mesh signed distances after treatment were 0.21 ± 0.11 mm for the maxillary teeth and 0.24 ± 0.05 mm for the mandibular teeth.^31^

### Skeletal changes of long‐term OA treatment

For the anteroposterior position of the maxilla, most studies did not find significant changes in SNA angle.^35^, ^36^, ^42^, ^47^, ^48^, ^51^, ^52^, ^57^, ^58^, ^60^, ^64^, ^65^, ^66^, ^69^, ^71^, ^73^ Robertson et al. observed a 0.32° increase in SNA after 30 months of OA use.^39^, ^40^ However, Alessandri‐Bonetti et al. found a 0.40° decrease in SNA angle after 3.5 years.^70^ Since the changes were small, these conflicting results could be ascribed to differences in the study (e.g., sample size) or appliance design (e.g., monobloc or bibloc). For the anteroposterior position of the mandible, seven studies observed a significant decrease of SNB angle,^35^, ^36^, ^47^, ^48^, ^51^, ^52^, ^56^, ^57^, ^65^, ^71^ while eight studies had no significance.^39^, ^40^, ^42^, ^58^, ^60^, ^64^, ^66^, ^69^, ^73^ Only one study by Geoghegan et al. demonstrated a 0.9° increase in SNB angle.^37^ However, the study used median and interquartile ranges to describe the data. For the relative position between maxilla and mandible, five studies observed a small but significant increase of the ANB angle,^35^, ^36^, ^39^, ^40^, ^47^, ^50^, ^65^ while nine studies did not find significant changes.^42^, ^48^, ^56^, ^57^, ^58^, ^64^, ^66^, ^67^, ^68^, ^69^, ^73^ For the vertical position of the maxilla and mandible, 6 out of 13 studies did find a significant increase of the MP‐SN angle,^33^, ^50^, ^56^, ^57^, ^65^, ^70^, ^73^ as well as all four studies investigating the MP‐FH angle showed a significant increase.^50^, ^56^, ^57^, ^58^, ^65^

In the meta‐analysis, the forest plots of SNA, SNB, ANB, MP‐SN, and MP‐FH, along with the mean difference and 95% CI are shown in Figure 6. The total change of SNA, SNB, and ANB was 0.10° (95% CI: ‐0.31–0.51, *p* = .62), 0.24° (95% CI: ‐0.18–0.67, *p* = .26), and ‐0.12° (95% CI: ‐0.40–0.16, *p* = .40), respectively. The total change of MP‐SN and MP‐FH angle was ‐0.55° (95% CI: ‐1.55–0.40, *p* = .28) and ‐1.21° (95% CI: ‐2.61–0.20, *p* = .09).

FIGURE 6The forest plots of skeletal changes in the long‐term OA treatment. (a) SNA. (b) SNB. (c) ANB. (d) MP‐SN. (e) MP‐FH. ANB, the sagittal relationship between the maxilla and mandible relative to the cranial base; OA, oral appliance; MP‐FH, the angle between the mandibular plane and the Frankfort plane; MP‐SN, (the angle between the mandibular plane and the cranial base), MP‐FH (the angle between the mandibular plane and the Frankfort plane; SNA, the sagittal relationship of the maxilla to the cranial base; SNB, the sagittal relationship of the mandible to the cranial base.

**Figure:**
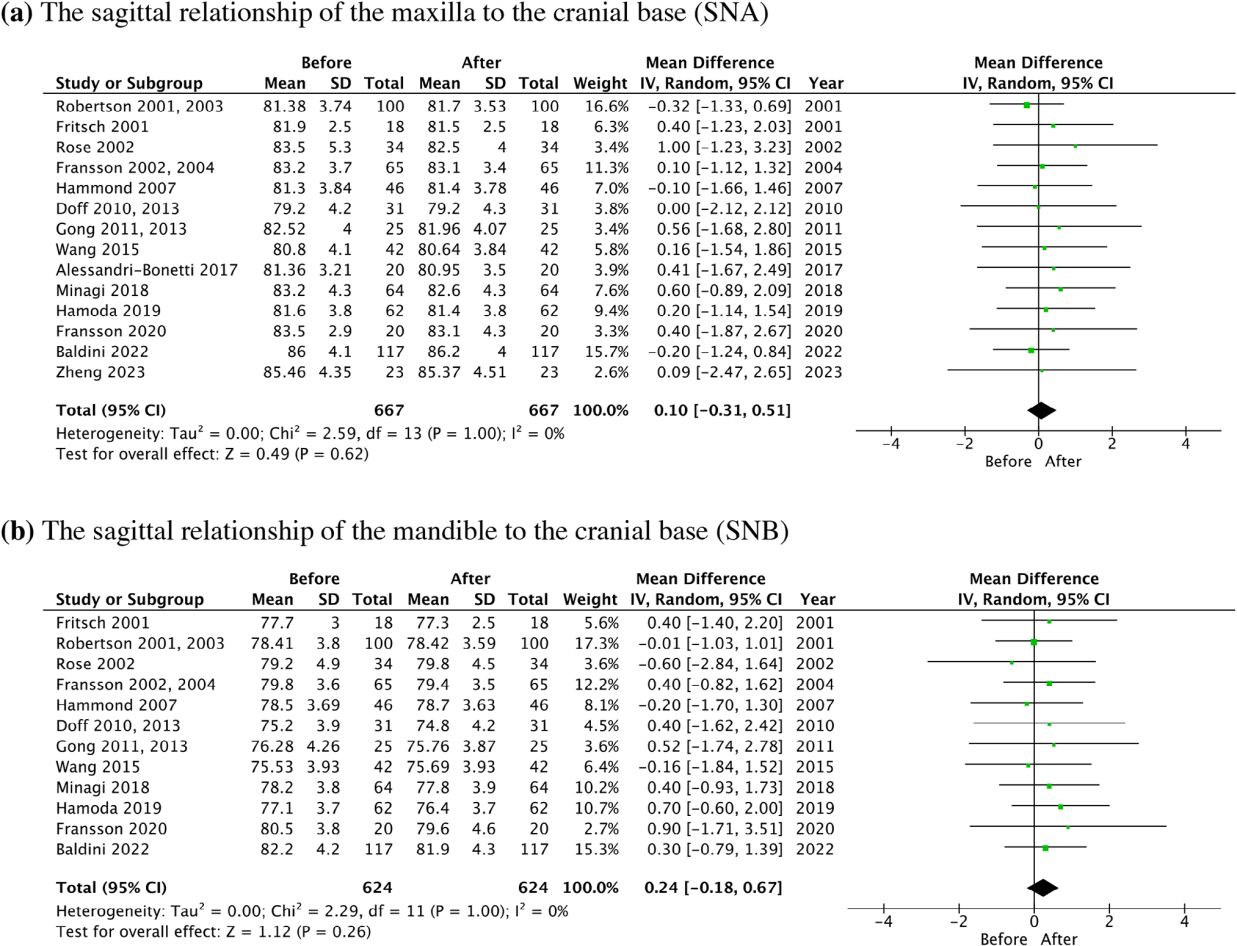

**Figure:**
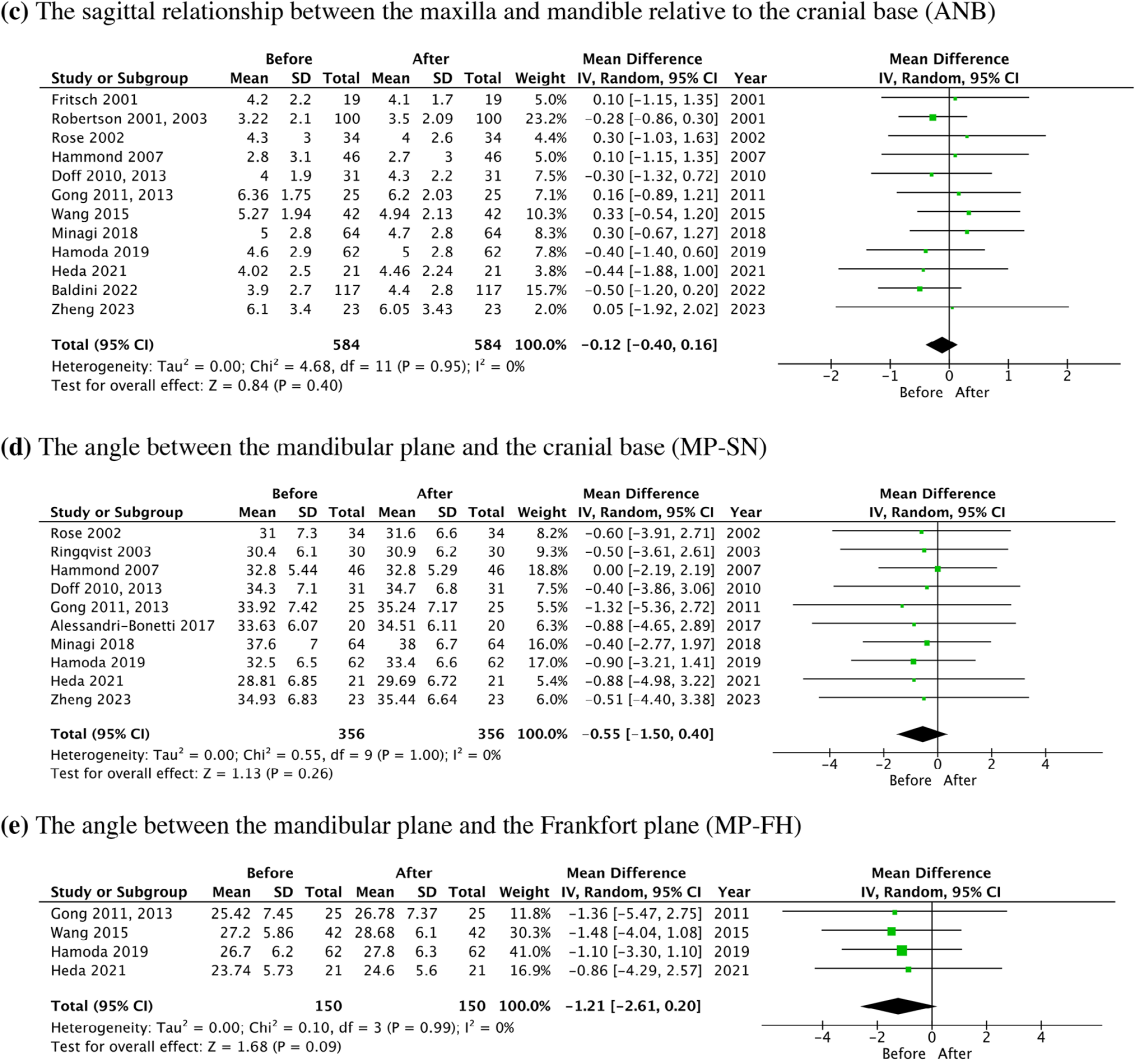

For TRDs, all three included studies performed dental cast analyses only without performing cephalometric analysis or investigating the skeletal changes. Therefore, evidence of skeletal changes from long‐term TRD use is lacking.

## DISCUSSION

This current systematic review and meta‐analysis, which included 42 studies in the systematic review and 23 in the meta‐analysis, found a significant decrease in OB and OJ, with a significant decrease in the U1‐SN angle and an increase in the L1‐MP angle in the long‐term OA treatment. All the variables indicating skeletal changes did not show a statistically significant difference over time.

The results of dental changes from different studies were quite consistent. The findings from individual studies, along with the meta‐analysis results, suggested that the decrease in OB and OJ was mainly due to the alternations of the axis angles of upper and lower incisors. According to the theory by Alessandri‐Bonetti et al.,^70^ when wearing the OA, the forward movement of the lower jaw can place a labially directed force against the crowns of mandibular incisors, thus increasing their proclination. The attached muscles, which are stretched to pull the mandible back, will simultaneously transmit the lingually directed force to the crowns of upper incisors, thus increasing their retroclination.

Compared with dental changes, the results of skeletal changes from different studies were less conclusive. For the anteroposterior position and the maxilla and mandible, several studies reached conflicting results. Along with the findings of the meta‐analysis, it suggested that the anteroposterior position of the maxilla and mandible might not change in the long‐term OA treatment. On the other hand, for the vertical position of the mandible and maxilla, as several studies had observed a significant increase of the MP‐SN and MP‐FH angle, as well as the pooled change of MP‐FH demonstrated a marginal statistical significance, there might be a tendency of the clockwise rotation of the mandible in the long‐term OA treatment. The downward rotation of the mandible could also partially explain the OB reduction and the decrease of SNB and ANB angle.

The mechanism of the vertical skeletal changes related to long‐term OA use is unclear. Wang et al. hypothesized that the downward rotation of the mandible was caused by the retroclination of the maxillary incisors and the proclination of the mandibular incisors through incisal guidance.^58^ From orthodontic literature, Bock et al. used Herbst devices in adults for Class II division 1 treatment for an average of 9 months.^74^ The mandible was moved forward by the bonded Herbst device, similar to the mechanism of MADs. The study found that the Class II malocclusion was corrected as a result of only tooth movement rather than the mandible being advanced or rotated.^74^

The systematic review also summarized the findings from three long‐term TRD studies. However, none of them observed significant dental changes over time. The possible reason is that the mechanism of TRDs is different from MADs.^30^ TRDs come into effect by maintaining the tongue at a protruded position with the suction force generated from a plastic bulb that is held between the lips and teeth.^75^ The protruded tongue could increase the volume of the upper airway, thereby improving airway patency and function.^76^ Unlike MADs that cover the whole dental arch and transmit forces directly into the teeth, the side effects of TRDs are mainly determined by altered tongue pressure.^77^ Since the tongue has a great capability to adapt to the different sizes and positions where sits, the dental changes caused by the tongue mainly result in dynamic arch expansion,^30^ and might not be as obvious as the changes caused by direct force from the MADs. However, unlike the studies of MADs, all three TRD studies had a small sample size and did not investigate the skeletal changes. These hypotheses need to be further evaluated and verified in well‐controlled, large‐scale prospective settings.

Overall, the findings from this study have several important clinical implications for the management of OSA patients undergoing OA treatment. First, clinicians should conduct a thorough baseline assessment of dental and skeletal parameters before initiating treatment. Additionally, ensuring that patients are fully informed about the potential long‐term side could help alleviate concerns and enhance treatment adherence. Throughout the treatment process, regular assessment of occlusal relationships and cephalometric measurements can help identify significant dental and skeletal alterations. For patients experiencing significant bite changes, interventions such as orthodontic correction may be necessary to ensure optimal treatment outcomes.

This study has several limitations. First, subgroup analyses revealed that heterogeneity in the included studies was mainly influenced by study design (retrospective vs. prospective), treatment duration (from 6 months to >10 years), and outcome measurements (dental casts, cephalometric analysis, and intraoral exams), emphasizing the need for adequately powered studies. Second, the majority of the included studies were assessed to have a serious risk of bias, particularly in the domains of measurement of outcomes and selection of participants, which led to a lower level of evidence. Third, most included studies did not take the drop‐outs into account. Several studies reported that side effects arising from long‐term OAT use, especially bite change, may lead to poor patient adherence.^78^, ^79^, ^80^, ^81^ Therefore, the ignorance of drop‐outs might cause the underestimation of the severity of bite changes.

In the future, larger‐scale, randomized controlled, and prospective studies with extended follow‐up periods are expected to better track the long‐term effects of OA therapy. Additionally, standardization of outcome measurements could further reduce heterogeneity and improve the reliability of future research. Other studies investigating the predictors and influence factors of dental and skeletal changes, such as patient demographics, OA design, and treatment adherence, are also expected to refine our understanding of treatment outcomes and provide evidence‐based practice guidelines.

## CONCLUSIONS

In the long‐term OA treatment, both OB and OJ gradually decreased over time, which might predominantly result from the retroclination of the upper incisors and the proclination of the lower incisors. Skeletal changes were not significant in the anteroposterior and vertical dimensions, suggesting that skeletal patterns might remain relatively stable. There is a tendency for the clockwise rotation of the mandible, which could probably contribute to the OB reduction and the posterior movement of the mandible.

## CONFLICT OF INTEREST STATEMENT

The authors declare no conflicts of interest.

## Supporting information

Supporting Information

## Data Availability

The data of this study are available from the corresponding author upon reasonable request.
