# Supplementary material for: Dentoskeletal changes of long‐term oral appliance treatment in patients with obstructive sleep apnea: A systematic review and meta‐analysis
Source: J Prosthodont. 2024 Sep 26;34(Suppl 1):62–79. doi: 10.1111/jopr.13946 (PMC12000640; doi:10.1111/jopr.13946)
Supplement: Supplementary file 1 — Supporting Information [file JOPR-34-62-s001.docx]

**Table S1a** Searching strategy for Ovid MEDLINE database.

| **#** | **Query** | **Results from April 1^st^, 2024** |
| --- | --- | --- |
| 1 | sleep apnea syndromes/ or sleep apnea, central/ or sleep apnea, obstructive/ | 43,912 |
| 2 | (osa or apn?ea*).tw,kf. | 66,504 |
| 3 | 1 or 2 | 73,453 |
| 4 | Occlusal Splints/ or Mandibular Advancement/ or orthodontic appliances/ | 13,767 |
| 5 | (MAS or mandibular advancement splint*).tw,kf. | 35,782 |
| 6 | (MAD or mandibular advancement device*).tw,kf. | 6,559 |
| 7 | (TRD or tongue retain*).tw,kf. | 2,659 |
| 8 | (TSD or tongue stabil*).tw,kf. | 1,728 |
| 9 | ((dental or occlusal or oral) adj3 (spint* or device* or appliance*)).tw,kf. | 4,365 |
| 10 | night?guard*.tw,kf. | 102 |
| 11 | or/4-10 | 63,318 |
| 12 | exp treatment outcome/ | 1,275,086 |
| 13 | ((adverse or negative or side or poor or worse or bad or safe* or undesir* or unwant*) adj3 (outcome* or effect* or event* or consequence* or manifest*)).tw,kf. | 1,301,157 |
| 14 | "treatment adherence and compliance"/ or patient compliance/ or medication adherence/ or "Duration of Therapy"/ | 86,686 |
| 15 | (non?complian* or non?adheren* or adheren* or complian*).mp. | 432,621 |
| 16 | (treatment duration or wearing time or wearing hour* or duration of treatment or duration of therap*).tw,kf. | 36,338 |
| 17 | or/12-16 | 2,751,549 |
| 18 | 3 and 11 and 17 | **1,195** |

**Table S1b** Searching strategy for Ovid Embase database.

| **#** | **Query** | **Results from April 1^st^, 2024** |
| --- | --- | --- |
| 1 | exp sleep disordered breathing/ | 14,365 |
| 2 | (osa or apn?ea*).tw,kf. | 108,735 |
| 3 | 1 or 2 | 113,156 |
| 4 | exp occlusal splint/ | 1,136 |
| 5 | mandibular advancement/ | 1,183 |
| 6 | exp orthodontic device/ | 23,841 |
| 7 | (MAS or mandibular advancement splint*).tw,kf. | 29,984 |
| 8 | (MAD or mandibular advancement device*).tw,kf. | 9,186 |
| 9 | (TRD or tongue retain*).tw,kf. | 4,149 |
| 10 | (TSD or tongue stabil*).tw,kf. | 2,412 |
| 11 | ((dental or occlusal or oral) adj3 (spint* or device* or appliance*)).tw,kf. | 5,222 |
| 12 | night?guard*.tw,kf. | 104 |
| 13 | or/4-12 | 75,088 |
| 14 | exp treatment outcome/ | 2,387,826 |
| 15 | ((adverse or negative or side or poor or worse or bad or safe* or undesir* or unwant*) adj3 (outcome* or effect* or event* or consequence* or manifest*)).tw,kf. | 195,0957 |
| 16 | patient compliance/ or medication compliance/ | 193,244 |
| 17 | treatment duration/ | 305,859 |
| 18 | (non?complian* or non?adheren* or adheren* or complian*).mp. | 701,861 |
| 19 | (treatment duration or wearing time or wearing hour* or duration of treatment or duration of therap*).tw,kf. | 67,399 |
| 20 | or/14-19 | 4,667,294 |
| 21 | 3 and 13 and 20 | **1,595** |

**Table S1c Searching strategy for Web of Science database.**

| **#** | **Query** | **Results from April 1^st^, 2024** |
| --- | --- | --- |
| 1 | TS=((osa or apn$ea*)) | 108,648 |
| 2 | TS=((MAS or mandibular advancement splint*)) | 64,032 |
| 3 | TS=((MAD or mandibular advancement device*)) | 22,519 |
| 4 | TS=((TRD or tongue retain*)) | 3,766 |
| 5 | TS=((TSD or tongue stabil*)) | 4,512 |
| 6 | TS=(((dental or occlusal or oral) NEAR/3 (spint* or device* or appliance*))) | 5,107 |
| 7 | TS=(night$guard*) | 52 |
| 8 | #2 OR #3 OR #4 OR #5 OR #6 OR #7 | 98,737 |
| 9 | TS=(((adverse or negative or side or poor or worse or bad or safe* or undesir* or unwant*) NEAR/3 (outcome* or effect* or event* or consequence* or manifest*))) | 1,492,126 |
| 10 | TS=((non$complian* or non$adheren* or adheren* or complian*)) | 535,729 |
| 11 | TS=((treatment duration or wearing time or wearing hour* or duration of treatment or duration of therap*)) | 356,146 |
| 12 | #9 OR #10 OR #11 | 2,273,100 |
| 13 | #1 AND #8 AND #12 | **687** |

**Table S4** Result of the studies that were excluded from meta-analysis, and the reasons for exclusion.

| **Author** | **Year** | **Study results** | **Reasons for Exclusion** |
| --- | --- | --- | --- |
| **Mandibular Advancement Devices (MADs)** | | | |
| Marklund^1^ | 2001 | MAD induced a change in overjet of -0.4 ± 0.8 mm and a change in overbite of -0.4 ± 0.7 mm. These changes were larger than those found in the reference group. | Outcome’s data were only reported as differences and median values. |
| Tegelberg^2^ | 2003 | None of the patients observed any changes in tooth contacts at intercuspidation after treatment. The difference of overbite showed no statistical significance. | Outcome’s data were only reported as mean and 95% confidence interval. |
| Battagel^3^ | 2005 | Results showed a median reduction in overjet of 0.4 mm (range 0.0-2.2) and a median reduction in overbite of 0.5 mm (range 0.6-2.0). These changes were significant at the 0.1% level. | Outcome’s data were only reported as median values. |
| Almeida^4,5^ | 2006 | Cephalometric analyses showed significant increases in mandibular plane and ANB angles; decreases in overbite and overjet; retroclined maxillary incisors; proclined mandibular incisors; increased lower facial height; and distally tipped maxillary molars with mesially tipped and erupted mandibular molars. | Outcome’s data were only reported as differences. |
| Marklund^6^ | 2006 | MADs induced a median change in overjet of -0.6 mm (range -3.5-1.3) and a change in overbite of -0.6 mm (range -5.0-1.3)  in frequent users. In infrequent users, the median change in overjet is -0.2 mm (range -1.6-1.5), and 0.0 mm (range -1.9-2.0) in overjet. | Outcome’s data were only reported as median values. |
| Hou^7^ | 2006 | The lower anterior facial height steadily increased over 3 years. A significant increase in the mandibular plane angle was also observed. Significant reductions in the overjet and overbite were observed only after 1-year, but not in 2 or 3-year follow up. | Outcome’s data were only reported as differences. |
| Marklund^8^ | 2010 | The mean changes in overjet were 0.1 mm for the orthodontic OA and -0.2 mm for the ordinary OA. The overbite decreased with the orthodontic OA (-0.57 mm), but not with the ordinary OA. The SNA angle decreased in the OA group, but was unchanged in the orthodontic OA group. | Outcome’s data were only reported as mean and range, but without standard deviation. |
| Vezina^9^ | 2011 | No statistical difference was found among the three groups. There was a trend toward a retroclination of the upper incisors and proclination of the lower incisors when comparing both treatment groups with the control group. | Outcome’s data were only reported as differences. |
| Geoghegan^10^ | 2015 | A significant reduction was found in the distances between the hyoid bone to retrognathia as well as the distance between the hyoid bone and mandibular plane angle. Soft palate length increased significantly. | Outcome’s data were only reported as median values. |
| Marklund^11^ | 2016 | Both the overjet and the overbite decreased significantly during OA treatment. The overjet changed between 0.1 and −5.0 mm, with a median value of −1.1 mm, while the overbite changed between 0.1 and −3.9 mm, with a median value of −1.6 mm. | Outcome’s data were only reported as differences and median values. |
| Norrhem^12^ | 2017 | The intercanine distances showed minor changes. The overjet was unchanged, while the overbite tended to decrease (-0.70 mm) without a statistical significance. | Outcome’s data were only reported as differences and median values. |
| Fransson^13^ | 2017 | Results showed significant decrease of overjet (−1.8 mm) and overbite (−1.5 mm). The mean change of both maxillary and mandibular intercanine width significantly decreased. | Outcome’s data were only reported as differences. |
| Teixeira^14^ | 2018 | There was a decrease in the overjet (-0.61 mm) and overbite (-0.76 mm) with significance. There was also an increase in the lower intercanine distance and L1-MP values. | Outcome’s data were only reported as differences. |
| Vigié du Cayla^15^ | 2019 | Results showed a modification of the inclination of the lower central incisors (+0.521) and of the position of the maxilla (0.287). | Outcome’s variables were different from any other studies. |
| Marklund^16^ | 2020 | Overjet (median -1.6 mm), overbite (median -0.7 mm), the molar relationship, and the irregularity of the lower front teeth had changed significantly during treatment. | Outcome’s data were only reported as median values. |
| Hu^17^ | 2020 | Results showed significant decrease of overjet (−0.42 mm) after an average of 6.57 y treatment. No significant change of overbite. Other dentition changes included intrusion of the upper premolars, buccalization of upper posterior teeth and mesialization of lower posterior teeth, decrease in upper arch length, increase in upper posterior arch width, and decrease in upper arch depth. | Outcome’s data were only reported as differences. |
| **Tongue Retaining Devices (TRDs)** | | | |
| Chen^18^ | 2008 | The most common appliance-induced dental changes included anterior and/or unilateral posterior open bite and reduced anterior overjet. | Outcome’s variables were not consistent and did not allow for systematic review. |
| Eid^19^ | 2016 | The TRD group did not reveal any significant changes from control group regarding overbite, overjet, and antero–posterior change in occlusion. | Outcome’s variables were not consistent and did not allow for systematic review. |
| Alshhrani^20^ | 2024 | Results demonstrated an overall enlargement of upper and lower arches. The average cloud-to-mesh signed distances after 1-year treatment were 0.21 ± 0.11 mm for the maxillary teeth and 0.24 ± 0.05 mm for the mandibular teeth. | Outcome’s data were only reported as differences. Outcome’s variables were not consistent and did not allow for systematic review. |

**Figure S1** Funnel plots of outcomes that has more than 10 eligible studies.

**(a)** Overbite

**(b)** Overjet

**(c)** The angle between the long axis of the lower central incisor and the mandibular plane (L1-MP)

**(d)** The sagittal relationship of the maxilla to the cranial base (SNA)

**(e)** The sagittal relationship of the mandible to the cranial base (SNB)

**(f)** The sagittal relationship between the maxilla and mandible relative to the cranial base (ANB)

**Figure S2** Subgroup analyses results

**(a)** Subgroup analysis on study design (retrospective vs. prospective)

OB

OJ

The test for subgroup differences suggested that there is a statistically significant subgroup effect (OB, *P* =.03; OJ, *P* =.007), indicating that study designs significantly affected the effect of OA treatment on OB and OJ. Prospective studies (OB, I^2^=4%; OJ, I^2^=17%) demonstrated more homogenous results compared to retrospective studies (OB, I^2^=35%; OJ, I^2^=48%).

**(b)** Subgroup analysis on risk of bias (low or moderate vs. high risk of bias)

OB

OJ

The test for subgroup differences indicates that there is no statistically significant subgroup effect (OB, *P* =.34; OJ, *P* =.31), suggesting that risk of bias did not modify the effect of OA treatment on OB and OJ. However, a smaller number of studies and participants contributed data to the low or moderate risk of bias subgroup than to the high risk of bias subgroup, indicating that the analysis may not be able to detect subgroup differences.

**(c)** Subgroup analysis on outcome measurements (dental cast analysis vs. cephalometric analysis vs. intraoral exams)

OB

OJ

The test for subgroup differences indicates that there is no statistically significant subgroup effect (OB, *P* =.11; OJ, *P* =.08), suggesting that outcome measurements not modify the effect of OA treatment on OB and OJ. However, studies conducting cephalometric analysis (OB, I^2^=0%; OJ, I^2^=0%) and intraoral exams (OB, I^2^=0%; OJ, I^2^=0%) demonstrated a marginally more homogenous results compared to studies using dental casts analysis dental casts analysis (OB, I^2^=65%; OJ, I^2^=74%).

**Reference**

1. Marklund M, Franklin KA, Persson M: Orthodontic side-effects of mandibular advancement devices during treatment of snoring and sleep apnoea. Eur J Orthod 2001;23:135-144

2. Tegelberg A, Walker-Engström ML, Vestling O, et al: Two different degrees of mandibular advancement with a dental appliance in treatment of patients with mild to moderate obstructive sleep apnea. Acta Odontol Scand 2003;61:356-362

3. Battagel JM, Kotecha B: Dental side-effects of mandibular advancement splint wear in patients who snore. Clin Otolaryngol 2005;30:149-156

4. Almeida FR, Lowe AA, Sung JO, et al: Long-term sequellae of oral appliance therapy in obstructive sleep apnea patients: Part 1. Cephalometric analysis. Am J Orthod Dentofacial Orthop 2006;129:195-204

5. Almeida FR, Lowe AA, Otsuka R, et al: Long-term sequellae of oral appliance therapy in obstructive sleep apnea patients: Part 2. Study-model analysis. Am J Orthod Dentofacial Orthop 2006;129:205-213

6. Marklund M: Predictors of long-term orthodontic side effects from mandibular advancement devices in patients with snoring and obstructive sleep apnea. Am J Orthod Dentofacial Orthop 2006;129:214-221

7. Hou HM, Sam K, Hägg U, et al: Long-term dentofacial changes in Chinese obstructive sleep apnea patients after treatment with a mandibular advancement device. Angle Orthod 2006;76:432-440

8. Marklund M, Legrell PE: An orthodontic oral appliance. Angle Orthod 2010;80:1116-1121

9. Vezina JP, Blumen MB, Buchet I, et al: Does propulsion mechanism influence the long-term side effects of oral appliances in the treatment of sleep-disordered breathing? Chest 2011;140:1184-1191

10. Geoghegan F, Ahrens A, McGrath C, et al: An evaluation of two different mandibular advancement devices on craniofacial characteristics and upper airway dimensions of Chinese adult obstructive sleep apnea patients. Angle Orthod 2015;85:962-968

11. Marklund M: Long-term efficacy of an oral appliance in early treated patients with obstructive sleep apnea. Sleep Breath 2016;20:689-694

12. Norrhem N, Nemeczek H, Marklund M: Changes in lower incisor irregularity during treatment with oral sleep apnea appliances. Sleep Breath 2017;21:607-613

13. Fransson AMC, Kowalczyk A, Isacsson G: A prospective 10-year follow-up dental cast study of patients with obstructive sleep apnoea/snoring who use a mandibular protruding device. Eur J Orthod 2017;39:502-508

14. Teixeira AOB, Andrade ALL, Almeida R, et al: Side effects of intraoral devices for OSAS treatment. Braz J Otorhinolaryngol 2018;84:772-780

15. Vigié du Cayla G, Collet JM, Attali V, et al: Long-term effectiveness and side effects of mandibular advancement devices on dental and skeletal parameters. J Stomatol Oral Maxillofac Surg 2019;120:7-10

16. Marklund M: Subjective versus objective dental side effects from oral sleep apnea appliances. Sleep Breath 2020;24:111-117

17. Hu JX, Xu SH, Mou SX, et al: [Three-dimensional model analysis of obstructive sleep apnea hyponea syndrome patients with long-term treatment of oral appliances]. Shanghai Kou Qiang Yi Xue 2020;29:202-207

18. Chen H, Lowe AA, Strauss AM, et al: Dental changes evaluated with a 3D computer-assisted model analysis after long-term tongue retaining device wear in OSA patients. Sleep Breath 2008;12:169-178

19. Eid H, Saifeldin H: Digital evaluation of changes in occlusion due to oral appliance therapy in obstructive sleep apnea. Egyptian Orthodontic Journal 2016;49:11-27

20. Alshhrani WM, Kohzuka Y, Okuno K, et al: Compliance and side effects of tongue stabilizing device in patients with obstructive sleep apnea. Cranio 2024;42:171-184
